# Inflammatory pseudotumor of eyelid: a probable IgG4-related sclerosing disease clinically mimicking eyelid pilomatrixoma

**DOI:** 10.1186/s12886-015-0004-4

**Published:** 2015-03-08

**Authors:** Youn Joo Choi, Min Joung Lee, Namju Kim, Ho-Kyung Choung, Sang In Khwarg, Ji Eun Kim

**Affiliations:** Department of Ophthalmology, Kangdong Sacred Heart Hospital, Hallym University Medical Center, Seoul, Korea; Department of Ophthalmology, Hallym Sacred Heart Hospital, Anyang, Korea; Department of Ophthalmology, Seoul National University Bundang Hospital, Seongnam, Korea; Department of Ophthalmology, Seoul Metropolitan Government Seoul National University Boramae Medical Center, 39, Boramae-gil, Dongjak-gu, Seoul, 156-707 Korea; Department of Ophthalmology, Seoul National University Hospital, Seoul, Korea; Department of Ophthalmology, College of Medicine, Seoul National University, Seoul, Korea; Department of Pathology, Seoul Metropolitan Government Seoul National University Boramae Medical Center, Seoul, Korea

**Keywords:** Eyelid, IgG4-related sclerosing disease, Ocular adnexa

## Abstract

**Background:**

Ocular adnexal IgG4-related sclerosing disease (IgG4-SD) has been categorized as a novel disease entity. It is characterized by stromal sclerosis and an infiltration of mass-forming lymphoplasmic cells containing many IgG4-positive plasma cells. Although ocular adnexal tissue involvement has been increasingly reported, a focal nodular sub-brow mass is not typical in an IgG4-SD presentation. We report a rare case of probable ocular adnexal IgG4-SD that clinically mimicked eyelid pilomatrixoma.

**Case presentation:**

A 42-year-old woman presented with a nodular mass in her left sub-brow area. The initial clinical impression of her lesion was eyelid pilomatrixoma. However, the final pathologic diagnosis was IgG4–SD, but extranodal marginal zone B-cell lymphoma could not be excluded. The patient underwent testing to determine tumor malignancy and systemic IgG4-SD involvement. Laboratory testing showed normal IgG and IgG4 serum levels and imaging revealed no remarkable findings. Oral prednisolone was administered and slowly tapered to manage the possible remnant lesion and to prevent disease recurrence. Two years after initiating therapy, there was no evidence of relapse. The patient is under close surveillance for signs of recurrence, systemic involvement, and potential malignant transformation.

**Conclusions:**

We found an unusual case of probable ocular adnexal IgG4-SD, which presented as a unilateral restricted mass involving the sub-brow area. Although the mass was surgically removed, systemic steroid treatment and long-term surveillance were initiated due to the possibility of recurrence, the potential association with systemic disease, and the potential development of extranodal mucosa-associated lymphoid tissue (MALT) lymphoma.

## Background

Ocular adnexal IgG4-related sclerosing disease (IgG4-SD) has been categorized as a novel disease entity that may account for a certain proportion of idiopathic ocular and periocular inflammatory lesions. It is characterized by stromal sclerosis and an infiltration of mass-forming lymphoplasmic cells containing many IgG4-positive plasma cells. It can involve various organs including pancreas, bile duct, retroperitoneal soft tissues, liver, thyroid, lung and salivary glands, either singly or systematically [1,2]. Although the prototype of IgG4-SD in ophthalmologic disorders is bilateral dacryoadenitis accompanied by sialadenitis (formerly termed Mikulicz disease), involvement of other ocular adenexal tissues such as orbital fat, extraocular muscles, and lacrimal sac has been increasingly reported in the literature [3-5]. However, a focal nodular sub-brow mass is not typical in an IgG4-SD presentation. We report a rare case of probable ocular adnexal IgG4-SD that clinically mimicked eyelid pilomatrixoma.

## Case presentation

**Figure 1:**
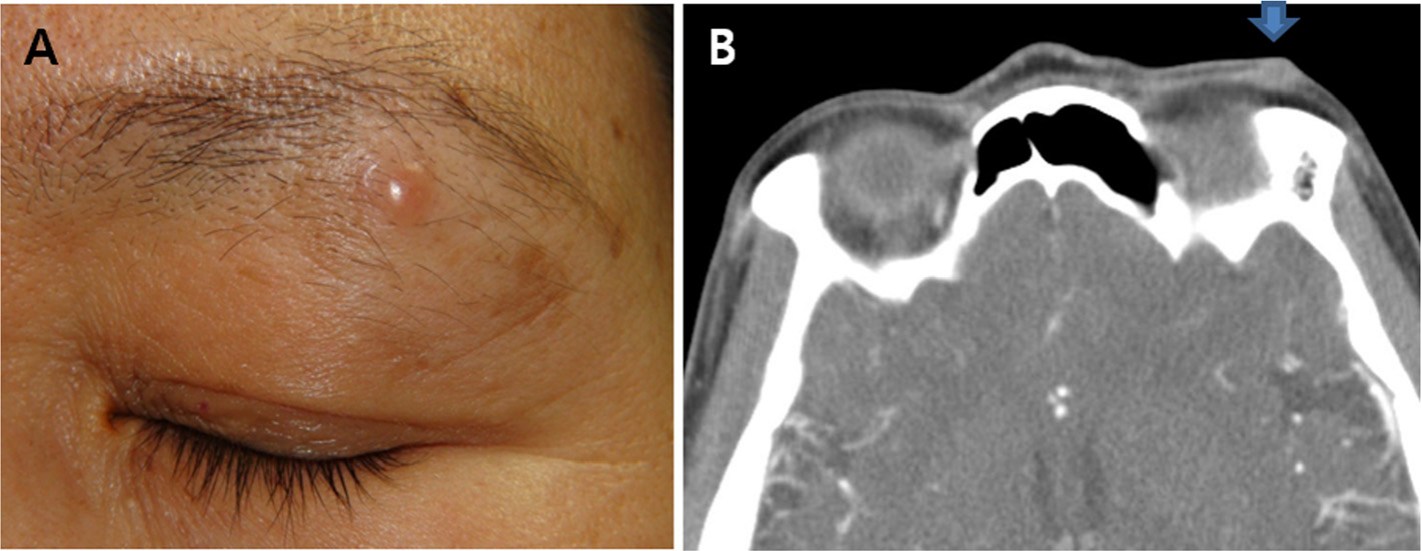
**Clinical presentation. (A)** Clinical photograph of a patient presenting with a 5.5 mm × 4.5 mm soft non-tender nodular mass with no overlying skin change. **(B)** An orbital CT scan showing a 7-mm nodular homogenous enhancing mass adherent to the skin of the left upper lid (arrow).

A 42-year-old woman with no significant past medical history presented with a painless nodular mass in her left upper lid, which had had slowly enlarged over 1 year. On clinical examination, a non-tender, nodular mass (5.5 mm × 4.5 mm) was palpable in the left sub-brow region and was freely mobile over subcutaneous tissue (Figure 1A). A diagnosis of presumed pilomatrixoma of the eyelid was made. Computed tomography with contrast of the orbit showed a 7-mm nodular homogenous enhancing mass that was adherent to the left upper lid skin (Figure 1B). This implied that the tumor had originated from either skin or a dermal appendage.

A total excisional biopsy was scheduled. Unfortunately, the tumor’s infiltrative growth pattern made it difficult to completely excise it from surrounding tissues. The tumor was pinkish to brown in color, poorly circumscribed, and had a rubbery surface.

**Figure 2:**
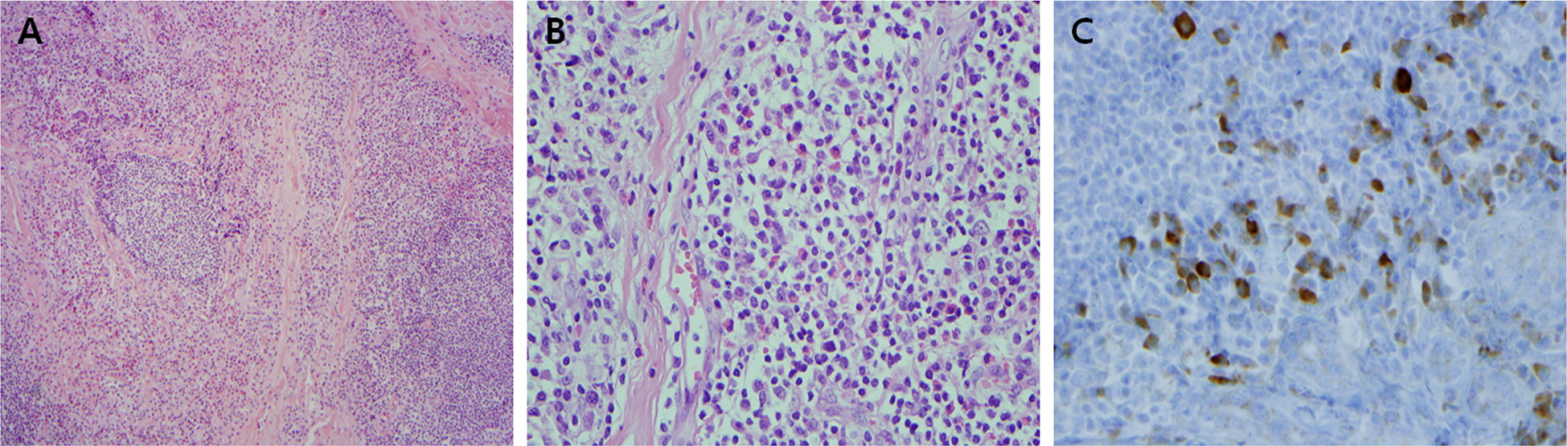
**Histopathology.** Biopsied specimen shows lymphoid nodules and interstitial fibrosis **(A)** hematoxylin-eosin staining, ×100 magnification). Under higher magnification, interfollicular spaces are filled with plasma cells and eosinophils **(B)** hematoxylin-eosin, magnification × 400). **(C)** Infiltration of IgG4-positive plasma cells numbered >50 in a high-power field (anti-IgG4 antibody, magnification × 400).

Histology revealed that the mass had a dense lymphocytic infiltration with lymphoid follicles, moderate plasma cell infiltration with a few eosinophils, and interstitial fibrosis (Figure 2A, B). Immunohistology showed increased CD20+ B-cells with slight marginal zone expansion, focal CD3+ T cell infiltration, a polyclonal plasma cell population with an even distribution of kappa and lambda light chains, and a low Ki-67 proliferative index (5% of all lymphoid cells). The number of IgG4+ plasma cells, counted in a high power field (HPF: X10 eyepiece and x 40 objective lenses), was >50 and the IgG4/IgG ratio was 50% (Figure 2C). The resulting final pathologic diagnosis was inflammatory pseudotumor with increased IgG4+ plasma cells; possible association with IgG4-SD, but extranodal marginal zone B-cell lymphoma could not be excluded.

Therefore, the patient underwent testing to determine tumor malignancy and systemic IgG4-SD involvement. Laboratory testing showed normal IgG and IgG4 serum levels. [1030 mg/dL (reference range, 700–1600), 21.1mg/dL (reference range, 6.1-121.4), respectively]. Serum Lactate dehydrogenase concentration was within normal range. Except for a 3 cm sized uterine myoma, imaging studies including chest CT, abdomen and pelvic CT, and positron emission tomography scanning demonstrated no remarkable findings.

For the management of possible remnant lesion and prevention of recurrence of disease, oral prednisolone was administered at a 40 mg/day initial dose and was slowly tapered to a 2.5 mg/day maintenance dose. One year after initiating therapy, there was no evidence of relapse. The patient is under close surveillance for signs of recurrence, systemic involvement, and potential malignant transformation.

## Discussion

Recognition of IgG4-SD is clinically important because this disease is, theoretically, a systemic disorder. Steroid therapy has been proposed to be effective, but relapse occurs frequently. Furthermore, recent studies suggest that IgG4+ plasma cells might be involved in the pathogenesis of mucosa-associated lymphoid tissue (MALT) lymphoma [6-8]. Moreover, many ocular adnexal MALT lymphoma cases are wrongly categorized as IgG4-SD, illustrating the difficulty of distinguishing between MALT lymphoma and IgG4-SD [7,9,10]. In our case, the lesion’s clinical appearance was typical of an eyelid pilomatixoma, a benign tumor originating from the hair follicle matrix that occurs frequently in the sub-brow area in the pediatric age group. Clinically, it appears as a slowly enlarging, non tender nodular mass which is freely movable over the subcutaneous tissue. The present case demonstrates the importance of pathologic confirmation, even when clinical examination suggests that a lesion is likely benign with clinically typical presentation.

Two characteristics IgG4-SD include dense sclerosis with abundant IgG4+ plasma cell infiltration and serum IgG4 level elevation [1]. In this case, as the clinical finding of unilateral focal subbrow mass was not typical presentation of IgG4-SD, the autoantibody profile including serum IgG4 level was not evaluated at initial presentation. After the pathologic confirmation of IgG4-SD, that is, two weeks after excisional biopsy, the postoperative serum IgG4 levels were checked. The timing of the measurement would be one of the possible explanations of the normal serum IgG4 level of this patient, as grossly no residual lesion existed postoperatively. Even though there was no preoperative evidence of serum IgG4 elevation in our patient, this case was given a probable diagnosis of IgG4-SD of the ocular adnexa based on the comprehensive diagnostic criteria for IgG4-SD (See the ‘Comprehensive diagnostic criteria for IgG4-related sclerosing disease’ section). It is true that serum IgG4 levels are frequently elevated in patients with IgG4-SD and this finding can be a useful diagnostic tool, however, not all of the reported cases with IgG4-SD revealed elevated serum IgG4 concentration [11-14]. Yamamoto et al. showed that high serum IgG4 levels are not specific to IgG4-SD [14].

### Comprehensive diagnostic criteria for IgG4-related sclerosing disease, 2011

1. Clinical examination showing characteristic diffuse/localized swelling or masses in single or multiple organs
2. Hematological examination shows elevated serum IgG4 concentrations( 135 mg/dl)
3. Histopathologic examination shows:
  1. Marked lymphocyte and plasmacyte infiltration and fibrosis.
  2. Infiltration of IgG4+ plasma cells: ratio of IgG4+/IgG+ cells > 40% and >10 IgG4 + plasma cells/HPF.

- Definite: 1) + 2) + 3)
- Probable: 1) + 3)
- Possible: 1) + 2)

## Conclusions

We found an unusual case of probable ocular adnexal IgG4-SD, which presented as a unilateral restricted mass involving the sub-brow area. Although the mass was surgically removed, systemic steroid treatment and long-term surveillance was initiated due to the possibility of recurrence, the potential association with systemic disease, and the potential development of extranodal MALT lymphoma.

## Consent

Written informed consent was obtained from the patient for publication of this Case Report and any accompanying images. A copy of the written consent is available for review by the Editor of this journal.

## Abbreviations

IgG4-SD: IgG4-related sclerosing disease
MALT: Mucosa-associated lymphoid tissue
HPF: High power field
